# A novel mouse model of diverse tumors derived from epithelial cells

**DOI:** 10.14814/phy2.70289

**Published:** 2025-03-28

**Authors:** Bin Wang, Lingxiang Wang, Yang Liu, Yasmin Jahan‐mihan, Gordon He, Yuxi Wang, Jie Hu, Jiale Wang, Yan Bi, Baoan Ji

**Affiliations:** ^1^ Department of Cancer Biology Mayo Clinic Jacksonville Florida USA; ^2^ Department of Gastroenterology Affiliated Drum Tower Hospital, Medical School of Nanjing University Nanjing China; ^3^ Department of Gastroenterology and Hepatology Mayo Clinic Jacksonville Florida USA

**Keywords:** CRISPR, Cytokeratin 19, Kras^G12D^, p53, tumors

## Abstract

A deeper understanding of pathophysiology, from the initiation of tumors of epithelial cells to the outcomes associated with it, is imperative to test potential interventions for these diseases. However, few models can accomplish this because of the complexity of origin cells and their transformation. We established an epithelial cell original tumor mouse model by conditional Kras^G12D^ activation and tumor suppressor gene p53 deletion specifically in epithelial cells of the whole body using Cytokeratin 19 (CK19) promoter via Cre‐ERT mediation. Multiple tumors derived from epithelial cells developed in this novel model. This model can recapitulate the progress of intestinal, pancreatic, liver, lung, and subcutaneous tumors which derive from epithelial cells. This model is suitable for studying the pathophysiology of tumors derived from epithelial cells and may have the potential to test interventions.

## Graphical abstract

CK19‐CreER/LSL‐KRas^G12D^/P53^L/L^/R26‐LSL‐LacZ mice could recapitulate multiple tumors originating from epithelial cells.

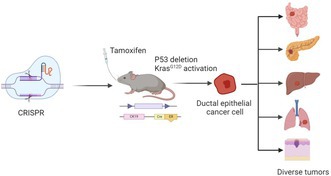

## 1 INTRODUCTION

Genetically engineered mouse (GEM) models are widely considered the most sophisticated animal models of human tumors. They are designed to mimic both genetic and pathologic aspects of cancer, which have been proven critical for drug development efforts, biomarker identification, and the study of early disease (Sharpless & Depinho, 2006; Tuveson & Hanahan, 2011). However, there is no ideal genetic model of multiple epithelial cell‐derived tumors for recapitulating the process or testing potential interventions for these diseases. Most GEM models can only induce epithelial cell tumors in a single organ, but no model can recapitulate multiple tumors originating from epithelial cells.

It has been reported that Cytokeratin 19 (CK19) belongs to a family of keratins, which are normally expressed in epithelial cells of the pancreas, liver, lung, and intestine etc. (Jain et al., 2010). Previous studies have used CK19 as a promoter to induce ICC (Ikenoue et al., 2016). It has also been shown that Kras and p53 mutations are two of the most frequent genetic events in human ICC and PDA, which are derived from ductal epithelial cells (Andersen et al., 2012; Nepal et al., 2018). Therefore, we generated compound mutant mice with CK19‐CreER‐mediated conditional activation of Kras^G12D^ and deletion of p53 in epithelial cells of the whole body. We found that Kras^G12D^ activation and homozygous p53 deletion in epithelial cells cooperate to induce intestinal, pancreatic, liver, lung, and subcutaneous tumors. So, in this study, we have successfully established a novel diverse tumor mouse model derived from epithelial cells.

## 2 MATERIALS AND METHODS

### 2.1 Construction of CK19‐CreER

Briefly, one guide was designed by visually confirming the protospacer adjacent motif (PAM) sequences of the exon 6 region of the mouse CK19 gene using Optimized CRISPR Design (http://crispr.mit.edu/). An IRES‐CreERT2 cassette was knocked into the mouse CK19 gene between the translation stop and 3‐UTR using the CRISPR/Cas9 technology (Ran et al., 2013).

### 2.2 Animals

The CK19‐CreER mice were developed by pronuclear injection into fertilized eggs derived from inbred C57BL/6J strain; R26‐LSL‐LacZ (https://www.jax.org/strain/003309) mice were from Jackson Laboratory (Lesche et al., 2002; Soriano, 1999). LSL‐KRas^G12D^/p53^L/L^ mice were obtained from NCI Mouse Repository (https://frederick.cancer.gov/resources/repositories/nci‐mouse‐repository/mousemodels/AvailableStrains.aspx) (Hingorani et al., 2003; Johnson et al., 2001; Jonkers et al., 2001). All transgenic mice were on the C57BL/6 genetic background. Both male and female mice were included in the study. All mice in this study are heterozygous for CK19‐CreER and LSL‐Kras^G12D^ and R26‐LSL‐LacZ alleles. All mice were housed on a 12‐h light/dark cycle in a temperature and humidity‐controlled room with food (PicoLab Rodent Diet 20, Irradiated, United States) and water. The animals were allowed to acclimatize for at least 7 days before the start of the experiment. The protocols of this study were approved by the Animal Care and Use Committee of Mayo Clinic.

### 2.3 Genotyping

Tail biopsies were performed at 2 weeks of age. DNA was prepared for PCR by incubating the tail with 250 μL of 50 mM sodium hydroxide at 100°C for 15 min, followed by neutralization with 25 μL of 1 M Tris (pH 8.0) containing 10 mM EDTA. PCR was carried out on the Eppendorf Mastercycler® X50S Thermocycler (Eppendorf, Hamburg, Germany). The PCR products were run on a 1–2% agarose gel stained with ethidium bromide. The PCR primers used for the genotyping of each mouse strain are listed in Table S1.

### 2.4 Experimental setup

X‐gal staining for β‐galactosidase was used for recombination efficiency and accuracy verification. Tamoxifen (TM; TRC‐T00600, 2923‐JPO‐240, LGC, United States) was given by gavage at 75 mg/kg for 5 days when the mice were 7 weeks old. Signs of esophagitis or aspiration from oral gavage will be closely monitored for 1 h. Any animals still having difficulty breathing or moving around the cage will be removed from the study. Animals that show signs of decline will be placed on heat support and IV fluids. If no improvement occurs after 4 h or the animal's condition worsens, they will be immediately euthanized and removed from the study. They will be euthanized via carbon dioxide (CO_2_) overdose. Tissues were harvested under anesthesia of isoflurane when the mice were lacking grooming, inactivity, cold to the touch, piloerection, squinted eyes, weight loss, and respiratory distress. Humane endpoints were based on veterinary clinical evaluation and Weight loss greater than or equal to 20% of body weight, inability to ambulate, inability to reach food and/or water, measuring 2 cm at the longest point of the single tumor. The cumulative tumor size (the sum of the longest point of each tumor) equaled 2 cm of multiple tumors.

### 2.5 Sacrifice and collection of samples

Animals were anesthetized with isoflurane, and fresh tissues of stomach, intestine, colon, lung, liver, and pancreas were collected. Each organ was divided into two parts for different processing methods. One part was fixed in fixation buffer for 5 h, followed by dehydration with 15% sucrose and 30% sucrose for 6 h separately, then embedded in OCT compound. The other part was fixed in 10% neutral buffered formalin and embedded in paraffin.

### 2.6 X‐gal staining for β‐galactosidase

Cryosections were cut at 6–10 μm in a cryostat at −20°C. Sections were fixed in X‐gal fixative containing 2% formaldehyde (F‐8857, MKCQ6645, Sigma‐Aldrich, United States), 0.2% glutaraldehyde (G‐6257, Sigma‐Aldrich, United States) in phosphate‐buffered saline (PBS) for 5 min on ice, followed by three washes with 1X PBS. X‐gal staining was performed in a solution containing 2 mg/mL X‐gal (BB0083, 1205BI607150, Bio Basic Inc., United States), 5 mM potassium ferrocyanide (PB131, WXBC 8601 V, Sigma‐Aldrich, United States), 5 mM potassium ferricyanide (P9387, WXBC 8448 V, Sigma‐Aldrich, United States), 1 mM MgCl2, and 0.02% NP‐40 in PBS (pH 7.5 for LacZ and pH 6.0 for senescence‐associate β‐galactosidase) at 37°C.

## 3 RESULTS

### 3.1 Development of CK19‐CreER mice

**FIGURE 1:**
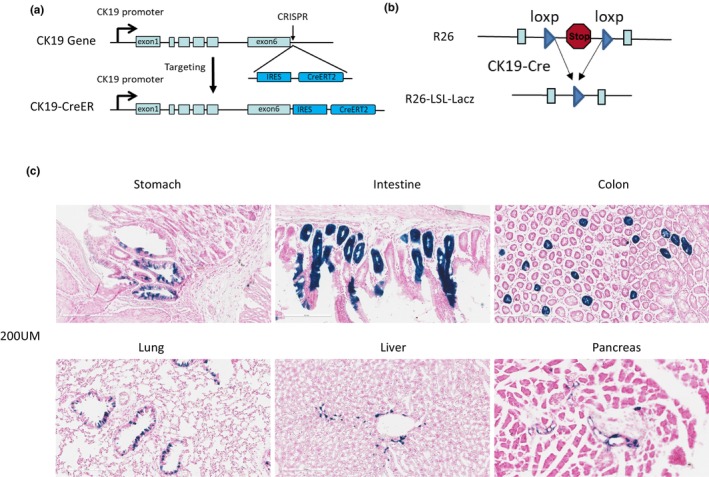
(a) Schematic drawing of CK19‐CreER transgenic mice generation. IRES‐CreERT2 was knocked in the mouse CK19 gene between translation stop and 3‐UTR by CRISPR. (b) Genetic strategy to conditionally activate reporter gene LacZ in epithelial cells of the whole body. CK19‐CreER mice were crossed with R26‐LSL‐LacZ mice. (c) β‐Galactosidase activity was examined by X‐gal staining in the tissue sections of the stomach, intestine, colon, lung, liver, and pancreas of the CK19‐CreER/R26‐LSL‐LacZ mice after tamoxifen treatment. Duct cells and epithelial cells are positively stained in these tissues.

The CRISPR‐modified gene CK19‐CreER was generated by knocking in the IRES‐CreERT2 cassette, and its insertion site and sequence were verified by DNA sequencing (Figure 1a). The CK19‐CreER mice were then produced through pronuclear injection. Furthermore, the CK19‐CreER/R26‐LSL‐LacZ mice were generated by inducing R26‐LSL‐LacZ mice (Figure 1b). CK19‐CreER induced genetic recombination in the epithelial cells of the whole body. Genotyping showed that CK19‐CreER and R26‐LSL‐LacZ of CK19‐CreER/R26‐LSL‐LacZ were all positive. Cre‐mediated LoxP recombination was confirmed by X‐gal staining of LacZ. Tissues of CK19‐CreER T/R26‐LSL‐LacZ were harvested 2 days after tamoxifen administration. X‐gal staining in the cryo‐sectioned tissues revealed that LacZ was expressed in the epithelial cells of the stomach, intestine, colon, lung, liver, and pancreas (Figure 1c).

Conditional Kras^G12D^ activation interacts with p53 deletion in epithelial cells, inducing diverse tumors.

**FIGURE 2:**
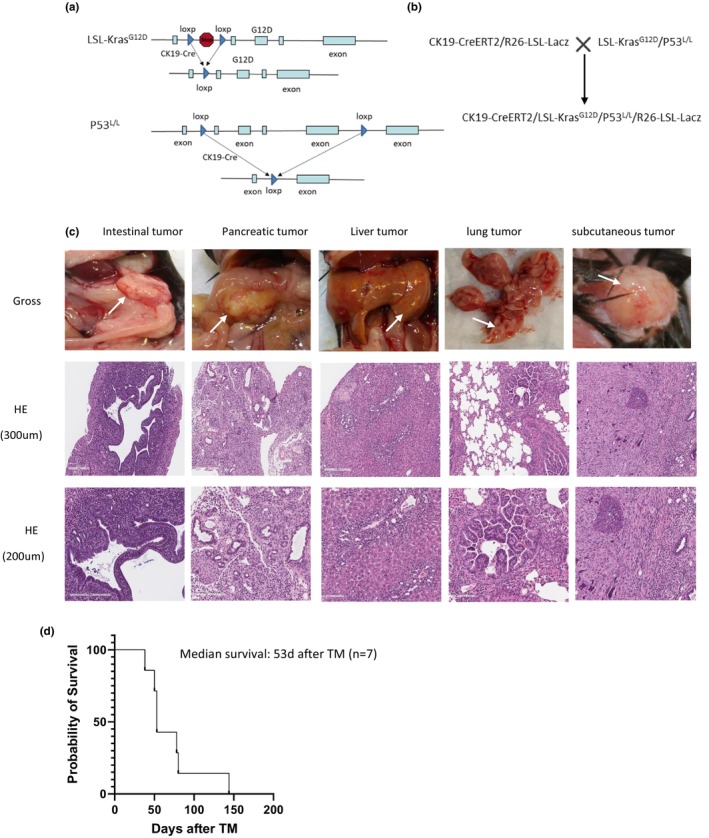
Characterization of the epithelial cell‐derived tumor mouse model. (a) genetic strategy to conditionally activate oncogenic Kras^G12D^ with homozygous p53 deletion in epithelial cells of the whole body. (b) Breeding strategy to generate CK19‐CreER/LSL‐KRas^G12D^/p53^L/L^/R26‐LSL‐LacZ mice. (c) Gross appearance and HE of tumors of CK19‐CreER/LSL‐KRas^G12D^/p53^L/L^/R26‐LSL‐LacZ mice. (d) Probability survival of CK19‐CreER/LSL‐KRas^G12D^/p53^L/L^/R26‐LSL‐LacZ mice after TM.

The CK19‐CreER/R26‐LSL‐LacZ mice were then crossed with LSL‐Kras^G12D^/p53^L/L^ mice to generate CK19‐CreER/LSL‐Kras^G12D^/p53^L/L^/R26‐LSL‐LacZ mice (Figure 2a,b). We also set CK19‐CreER/LSL‐Kras^G12D^/p53^L/+^/R26‐LSL‐LacZ, CK19‐CreER/LSL‐Kras^G12D^/R26‐LSL‐LacZ, and CK19‐CreER/p53^L/L^/R26‐LSL‐LacZ mice as controls. Genotyping showed that CK19‐CreER, LSL‐Kras^G12D^, p53^L/L^, p53^L/+^, and R26‐LSL‐LacZ were all positive. Most of these CK19‐CreER/LSL‐Kras^G12D^/p53^L/L^/R26‐LSL‐LacZ mice started to demonstrate distress at around 50 days after tamoxifen administration (Figure 2d), but the control mice had no signs of illness even 7 months after tamoxifen administration. Autopsies revealed that all the control mice had no tumors, but five of the seven CK19‐CreER/LSL‐Kras^G12D^/p53^L/L^/R26‐LSL‐LacZ mice had lung tumors, pancreatic tumors, and liver tumors; one mouse had an intestinal tumor, lung tumor, and pancreatic tumor, and another mouse had a pancreatic tumor, liver tumor, and subcutaneous tumor (Table 1), but none of them had stomach or colon tumors. Histology showed that tumor cells of the intestinal tumor were from epithelial cells and exhibited glandular structures, with disorganized cell arrangement and disrupted tissue architecture, and the cell nuclei appeared enlarged, with increased atypia and prominent nucleoli. The main features of lung tumors are nuclear enlargement, increased nucleoli, and coarsened chromatin. It showed that these tumor cells were from bronchial mucosal epithelium. These pancreatic tumors are PDA and typically present as irregular, branched, or tubular glandular structures. These glands are lined with irregularly arranged cancer cells, whose nuclei often exhibit pleomorphism and hyperchromasia. These liver tumors are ICC and typically present as irregular glandular structures lined by cuboidal or columnar epithelial cells, which exhibit marked nuclear atypia. The cell morphology of the subcutaneous tumor is irregular, with marked atypia, large and hyperchromatic nuclei, frequent mitotic figures, and disorganized cell arrangement (Figure 2c).

**TABLE 1:** Numbers and sites of tumors of CK19‐CreER/LSL‐Kras^G12D^/P53^L/L^/R26‐LSL‐LacZ mice.

Sites of tumors	Number of mice
Lung tumor	6
Pancreatic tumor	6
Liver tumor	6
Intestinal tumor	1
Subcutaneous tumor	1

## 4 DISCUSSION

In this study, we generated CK19‐CreER mice which mediate gene recombination in epithelial cells of the whole body via tamoxifen. The X‐gal staining of CK19‐CreER/R26‐LSL‐LacZ mice showed that it could target epithelial cells efficiently. Pioneering studies have reported the first GEM model with combined Kras^G12D^ activation and p53 deletion mediated by albumin‐Cre, and most of these GEM mice developed stroma‐rich CCA (O'Dell et al., 2012). Another study also showed that most CCA development was observed when Kras^G12D^ activation was combined with p53 deletion in the adult ductal compartment upon expression of Sox9‐driven Cre‐recombinase (Hill et al., 2018). However, CK19 and Sox9 are also expressed in intrahepatic cholangiocytes and epithelial cells in many other organs (Ikenoue et al., 2016; Lin et al., 2018). So, we generated CK19‐CreER/LSL‐Kras^G12D^/p53^L/L^/R26‐LSL‐LacZ mouse line and induced multiple epithelial cell‐derived tumors which we called diverse tumors. This novel mouse model could recapitulate multiple tumors that originated from epithelial cells uniquely, including intestinal epithelioma, PDA, CCA, bronchial lung tumor, and subcutaneous tumor.

Although some models developed thus far have considerably helped us to better understand important aspects of single epithelial cell tumors such as CCA or PDA, there has been no single model that could adequately recapitulate all hallmarks of epithelial cell‐derived tumors till now. Our tumor model recapitulated many hallmarks of epithelial cell‐derived tumors that cannot be modeled using in vitro systems or by xenotransplantation of human tumor cell lines or patient‐derived tissues (PDXs) into immunodeficient mice. Our model could help pre‐clinical platforms address prevailing questions including the characterization of the initial steps during cholangiocarcinogenesis, the dissection of histopathological and molecular features, and response and resistance to novel therapeutics of different tumors.

However, our model has several limitations. Though CK19‐CreER/LSL‐Kras^G12D^/p53^L/L^/R26‐LSL‐LacZ mice could generate the intestinal epithelioma, PDA, CCA, bronchial lung tumor, and subcutaneous tumor, we did not find gastric or colon tumors generated. This may be due to the varying sensitivity of different tissues to oncogenes and tumor suppressor genes. Additionally, Cre recombination efficiency in the colon and stomach exhibits a mosaic pattern, potentially leading to incomplete recombination and insufficient oncogenic activation in these tissues. Furthermore, previous studies have reported that Kras mutations occur in only 10–15% of gastric cancer cases but are found in approximately 30–50% of colorectal tumors, which may contribute to the observed differences in tumor formation (Won & Choi, 2022). Another limitation of our model is that, although it can induce diverse tumors, it cannot generate multiple tumor types simultaneously due to tumor heterogeneity.

In summary, we have successfully established a novel mouse model that could recapitulate multiple tumors originating from epithelial cells uniquely. This mouse model should provide a better understanding of tumors derived from epithelial cells and facilitate the development of new therapies for the same type of diseases.

## AUTHOR CONTRIBUTIONS

B.W. performed most of the experiments and drafted the manuscript. Y.L., L.W., Y.J., Y.W., and J.H. performed experiments; J.W., Y.B., and G.H. edited and revised the manuscript. B.J. supervised the study, secured funding, and finalized the manuscript.

## FUNDING INFORMATION

This study was supported by the Mayo Clinic Foundation startup fund, the Mayo Clinic Center for Biomedical Discovery (to B.J.), and the Mayo Clinic Hepatobiliary SPORE P50 CA210964 (to B.J.).

## CONFLICT OF INTEREST STATEMENT

No conflicts of interest, financial or otherwise, are declared by the authors.

## Supporting information


Data S1.

## Data Availability

The datasets and mice generated in this study are available from the corresponding author upon request.
