# Supplementary material for: A novel mouse model of diverse tumors derived from epithelial cells
Source: Physiol Rep. 2025 Mar 28;13(7):e70289. doi: 10.14814/phy2.70289 (PMC11950630; doi:10.14814/phy2.70289)
Supplement: Supplementary file 1 — Data S1. [file PHY2-13-e70289-s001.docx]

Supplementary Table 1. List of Primers used in this study.

| Genes | Forward primers | Reverse primers |
| --- | --- | --- |
| CK19-CreER | cacggggacgtggttttcct | gttgcatcgaccggtaatgca |
| LSL-KRas^G12D^ | ccatggcttgagtaagtctgc | cgcagactgtagagcagcg |
| p53^L/L^ | cacaaaaacaggttaaacccag | agcacataggaggcagagac |
| R26-LSL-LacZ | atcctctgcatggtcaggtc | cgtggcctgattcattcc |
